# Transcaval access for transcatheter aortic valve replacement: a systematic review and meta-analysis

**DOI:** 10.1097/MS9.0000000000003193

**Published:** 2025-03-28

**Authors:** Zina Otmani, Ahmad Alzawahreh, Abdelaziz A. Awad, Basma Badrawy Khalefa, Hazem Ayman Elsayed, Omar Hany Mohamed Amin, Mohamed Abouzid, Amjad Almansi, Ayman K. Awad

**Affiliations:** a Faculty of Medicine, Mouloud Mammeri University, Tizi Ouzou, Algeria; b Faculty of Medicine, The Hashemite University, Zarqa, Jordan; c Faculty of Medicine, Azhar university, Cairo, Egypt; d Faculty of Medicine, Ain-Shams University, Cairo, Egypt; e Faculty of Medicine, Zagazig university, Egypt; f Department of Physical Pharmacy and Pharmacokinetics, Faculty of Pharmacy, Poznan University of Medical Sciences, Poznan, Poland; g Department of Internal Medicine, Prince Hamza Hospital, Amman, Jordan

**Keywords:** meta-analysis, transcatheter aortic valve replacement, transcaval

## Abstract

**Background:**

Transfemoral access is considered the standard route for transcatheter aortic valve replacement (TAVR). However, in some cases, this access route is contraindicated. Alternative access routes, such as transaxillary, are emerging as first-line approaches to address these limitations, while the transcaval approach remains uncertain. Our Meta-analysis aims to evaluate the safety of the transcaval approach compared to other access routes for TAVR.

**Methods:**

We searched five databases, PubMed, SCOPUS, Web of Science, Cochrane Library, and Embase, from inception until April 2024 for any study that reports transcaval access alone or compares it to any alternative access in TAVR patients. We used Risk ratio (RR) for dichotomous outcomes and Mean Difference (MD) for continuous outcomes with a 95% confidence interval.

**Results:**

Eleven studies with 1003 patients were included, transcaval showed non-statistically significant difference over alternatives in terms of intra-hospital, 30 days, and long-term all-cause mortality with a RR of 0.08 (95% CI −0.83 to 0.99, *P* = 0.86), 0.11 (95% CI −0.48 to 0.7, *P* = 0.71), and 1.08 (95% CI −0.50 to 2.66, *P* = 0.18), respectively. Furthermore, in our single-arm analysis, transcaval access has a low pooled 30-day all-cause mortality rate of 7% (95% CI 5–10) and long-term mortality of 21% (95% CI 2–77). The mean difference in length of stay in the hospital after the operation was 4.88 (95% CI 4.45–5.31), and the major bleeding rate was 14% (95% CI 8–23).

**Conclusion:**

Transcaval access for TAVR is a safe and feasible method that can be considered a new alternative when trans-femoral access is contraindicated.

## Introduction

Aortic stenosis (AS) is the most common primary valvular heart disease, with a rapid increase in its prevalence with aging^[1,2]^. Transcatheter aortic valve implantation (TAVI) is a class I recommendation according to ESC/EACTS Guidelines^[1]^ in older patients >75 years with high surgical risk or intermediate surgical risk with favorable clinical, anatomical, and procedural characteristics which are decided by the heart team. Furthermore, several studies have been focused on the role of TAVI in younger and low-risk patients with severe aortic stenosis^[3]^.

**HIGHLIGHTS**

- Transcaval access showed no statistically significant difference in intra-hospital, 30-day, and long-term all-cause mortality compared to alternatives.
- The single-arm analysis indicated a low 30-day all-cause mortality rate of 7% and a long-term mortality rate of 21%.
- Transcaval access for transcatheter aortic valve replacement is a safe and feasible alternative, especially when trans-femoral access is not an option.

The transfemoral approach is the primary preferable access for TAVI. However, for some patients with abnormal vascular anatomy or peripheral arterial disease who are unsuitable for a transfemoral approach, alternative percutaneous approaches such as transaxillary, trans-subclavian, transcarotid, and transcaval approaches are considered^[4]^. The invasive surgical approaches like transapical or transaortic access are less likely to be considered recently because of their poor outcomes^[5]^. Thus, the trans-subclavian approach is the second-line access after the transfemoral one. However, it is contraindicated in abnormal arterial anatomy, severe angulation, stenosis, and using the internal mammary artery grafts in coronary artery bypass graft surgery^[6]^.

The transcaval approach is the latest TAVI technique, which allows larger introducer sheaths through the inferior vena cava to the infrarenal aorta, bypassing the abnormal iliofemoral arteries. At the end of the procedure, the aortic hole is closed with a nitinol occluder without a higher risk of major bleeding^[7]^. Recent studies imply that the transcaval approach could be the first alternative access for the transfemoral approach as it shows better outcomes than subclavian access^[8]^. Therefore, we conducted our meta-analysis to gain insight into the safety and efficacy of transcaval access and compare it to the other alternative accesses for TAVI when transfemoral access is contraindicated.

## Methods

Our systematic review and meta-analysis strictly adhered to the Preferred Reporting Items for Systematic Reviews and Meta-Analyses (PRISMA) guidelines^[9]^. The methodology was meticulously designed and executed following the standards defined in the Cochrane Handbook for Systematic Reviews of Interventions^[10]^ to ensure methodological rigor and transparency. Furthermore, this study has been officially registered on the Open Science Framework under the DOI: 10.17605/OSF.IO/WUASM.

### Literature search and study selection

We systematically and comprehensively searched multiple electronic databases, including PubMed, Scopus, Web of Science, the Cochrane Central Register of Controlled Trials, and Embase, covering all records from inception until 22 April 2024. The search strategy was designed using a predefined query to ensure the inclusion of relevant studies:

(((Transcatheter Aortic Valve Implantation) OR (TAVI) OR (transcatheter aortic valve replacement) OR (TAVR)) AND ((Transcaval) OR (caval-aortic access)) AND ((nonfemoral access)) OR (alternative access) OR (Transcarotid access) OR (Trans-Subclavian access) OR (Transaxillary) OR (Transapical))).

Duplicate records were identified and removed using EndNote (Clarivate Analytics, PA, USA). The screening process was carried out in two distinct stages. In the first stage, two independent reviewers screened the titles and abstracts of all retrieved articles to evaluate their relevance to this meta-analysis. In the second stage, full-text articles of potentially eligible studies were thoroughly reviewed to determine their final inclusion. Any discrepancies between reviewers were resolved through discussion, and if consensus could not be reached, a third reviewer was consulted to make the final decision.

### Eligibility criteria

We included all studies that report transcaval access alone or compare it to any alternative access in TAVR patients. We included all studies following these criteria:

1. *Population*:

a. any patients above 18 years old

b. had severe symptomatic aortic stenosis with an indication for TAVR

c. With one or several contraindications to the gold standard transfemoral vascular access who underwent TAVR using transcaval vascular access.

2. *Intervention*: Transcaval TAVR
3. *Comparator*: any alternative access in TAVR patients
4. *Outcome:*
  a. *Primary outcomes: mortality, including hospital stay or 30 days of all-cause mortality*, and 1-year mortality.
  b. *Secondary outcomes*: Morbidity including intra-hospital stay, stroke, vascular complications, major bleeding, acute kidney injury, new pacemaker implantation, readmission within 30 days, and blood transfusion.
5. *Study design*: any prospective or retrospective cohort observational studies.

We excluded case reports, case series, conference papers, letters to the editor, posters, unpublished articles, and animal studies.

### Data extraction and quality assessment

The baseline characteristics were extracted from the included studies, such as first author, publication year, sample size, sex, mean age, follow-up duration, left ventricular ejection fraction, hypertension, diabetes, previous coronary artery bypass graft surgery, prior stroke, prior renal failure or dialysis, lung disease, peripheral vascular disease. Furthermore, for the analysis, we extracted hospital or 30 days all-cause mortality and 1-year mortality, secondary outcomes: Morbidity including intra-hospital stay, stroke, vascular complications, major bleeding, acute kidney injury, new pacemaker implantation, readmission within 30 days, and blood transfusion, heart failure in 6 months to 1 year.

The methodological quality of the included studies was rigorously assessed using standardized evaluation tools to ensure reliability and validity. For observational pairwise studies, the Newcastle-Ottawa Scale (NOS)^[11]^ was employed, which evaluates studies based on key domains, including selection, comparability, exposure, and outcome measures. Additionally, the quality of single-arm studies was appraised using the National Institutes of Health (NIH) Quality Assessment Tool, providing a comprehensive evaluation of study design and methodological robustness.

### Data analysis

We conducted a pairwise meta-analysis using STATA (version 17)^[12]^ and a single-arm meta-analysis using R version 4.3.3 software^[13]^. Considering a *P* value less than 0.05 as statistically significant, fixed-effects models were employed if there was no significant heterogeneity, and random-effects meta-analysis models were employed if there was a significant heterogeneity. Continuous data was reported in mean and standard deviation (SD), while dichotomous data was reported in proportions or rates in single arm or risk ratio (RR) in pairwise meta-analysis. All data were calculated with a 95% confidence interval (CI) to assess the estimated effect measure. Moreover, heterogeneity was evaluated using the inconsistency (I^2^) and Chi-squared (X^2^) tests. I^2^ >50% was considered substantial heterogeneity in the studies. If substantial heterogeneity was found, a sensitivity analysis was performed to show how each study affects the overall estimate by excluding one study interchangeably from the meta-analysis.

## Results

### Literature search results

The research strategy we used resulted in a total number of 928 studies. A total of 430 studies were removed as duplicates. After the title and abstract screening, 29 full-text articles were evaluated for eligibility. Full-text screening resulted in 11 studies^[7,14-22]^ that met our criteria and were included in our meta-analysis with all observational studies (Supplementary Figure 1, available at: http://links.lww.com/MS9/A775).

### Characteristics of the included studies

A total of 1003 patients were included in our study, with a mean age of 79.3 years. Females were 548 (54.6%) of the included patients, with trans-subclavian being the most compared technique to transcaval. Further details about baseline characteristics and a summary of included studies can be found in Table 1. After applying the NOS for the quality assessment of our five observational studies, all the studies were shown to be of good quality. Moreover, upon using the NIH assessment tool for assessing our single-arm studies, all of them showed a low risk of bias in all domains except for sample size justification, which showed a high risk of bias, and exposure variability, which showed unclear risk. Further details can be found in Supplementary Figures 2–5 (available at: http://links.lww.com/MS9/A775).

**Table 1:** Table of baseline characteristics

Study	Number of arms	Access for TAVR	Number of patients, n	Follow up duration, days	Age, years, mean ± SD	Sex female, n(%)	Previous CABG, n(%)	Prior MI, n(%)	Prior stroke or cerebrovascular disease, n(%)	Diabetes, n(%)	Hypertension, n(%)	Atrial fibrillation, n(%)	Renal failure or dialysis n(%)	LVEF %, mean ± SD	Mean AVPG, mean ± SD	Lung disease, n(%)
Barbash 2021	Double arm study	TC	20	30 days	82 ± 3.1	5 (25%)	7 (35%)	95 (58%)	2 (10%)	10 (50%)	17 (85%)	7 (35%)	12 (60%)	51 ± 15.95	38.33 ± 21.54	2 (10%)
		Alternative access	165		82 ± 6.73	97 (48%)	50 (30%)	33 (20%)	30 (18%)	60 (36%)	139 (84%)	32 (19%)	122 (74%)	56 ± 10.47	40.66 ± 14.95	25 (15%)
Costa 2020	Single arm study	TC	50	30 days	78.7 ± 8.0	29 (58%)	14 (28%)	15 (30%)	7 (14%)	13 (26%)	44 (88%)	20 (40%)	30 (60%)	13 (26%)	39 ± 15	18 (36%)
Grazina 2023	Double arm study	TC	10	1 year	78.9 ± 7.0	9 (27%)	5 (15%)	6 (18%)	8 (24%)	12 (35%)	NA	14 (41%)	21 (62%)	NA	47 ± 14	NA
		TS	24													
Greenbaum 2014	Single arm study	TC	19	111 ± 57 days	80.7 ± 8.3	15 (79%)	NA	NA	NA	NA	NA	10 (53%)	NA	NA	NA	NA
Greenbaum 2017	Single arm study	TC	99	30 days	79.16 ± 9.02	58 (58%)	NA	NA	NA	NA	NA	NA	10 (10%)	52.8 ± 15.6	NA	NA
Lederman 2019	Single arm study	TC	99	365 days	79.33 ± 9.02	57 (58%)	NA	NA	NA	NA	NA	NA	10 (10%)	52.6 ± 15.6	NA	NA
Lederman 2022	Double arm study	TC	238	365 days	76.4 ± 9.1	134 (56.3%)	64 (26.9%)	58 (24.4%)	52 (21.8%)	90 (37.8%)	223 (94.1%)	82 (34.6%)	21 (8.8%)	53.66 ± 12.67	40.1 ± 13.5	96 (40.3%)
		TA	106		77.2 ± 8.8	46 (43.4%)	21 (19.8%)	27 (25.7%)	26 (24.5%)	46 (43.4%)	98 (92.5%)	36 (34.0%)	6 (5.7%)	54.3 ± 15.03	41.1 ± 13.9	35 (34.0%)
Long 2020	Double arm study	TC	22	356 days	80.7 ± 3.9	14 (63.6%)	3 (13.6%)	6 (27.3%)	4 (18.1%)	7 (31.8%)	NA	5 (22.7%)	2 (9%)	42.3 ± 4.2	38.2 ± 4.8	5 (22.7%)
		TS	41		83.2 ± 3.7	24 (58.5%)	4 (9.8%)	5 (12.2%)	6 (14.6%)	18 (43.9%)	NA	11 (26.8%)	3 (7.3%)	38.3 ± 5.9	34.8 ± 4.7	10 (24.3%)
Michail 2022	Single arm study	TC	8	132 ± 452 days	70.3 ± 9.8	6 (75.0%)	NA	1 (12.5%)	2 (25%)	3 (37%)	NA	2 (25%)	NA	NA	46.4 ± 21.4	••
Paone 2018	Double arm study	TC	58	30 days	79.6 ± 9.6	32 (55.2%)	NA	NA	25 (43.1%)	26 (44.8%)	52 (90.0%)	NA	3 (5.2%)	53.3 ± 15.5	32.0 ± 13.1	25 (43.1%)
		TCc	32		79.0 ± 9.6	16 (50.0%)	NA	NA	13 (40.6%)	11 (34.4%)	30 (93.8%)	NA	2 (6.3%)	0.562 ± 0.119	36.5 ± 15.2	20 (62.5%)
Rogers 2019	Single arm study	TC	12	30 days	81.6 ± 10.9	6 (50%)	3 (25%)	NA	1 (12%)	3 (25%)	10 (83%)	2 (17%)	NA	NA	••	3 (25%)

NA: not available; SD: standard deviation; TC: transcaval; TS: trans-subclavian; TA: transaxillary; TCc: transcarotid; CABG: coronary artery bypass grafting; MI: myocardial infarction; AVPG: aortic valve pressure gradient.

### Meta-analysis

#### Pairwise analysis

- Mortality
  - Intra-hospital death

**Figure 1.**
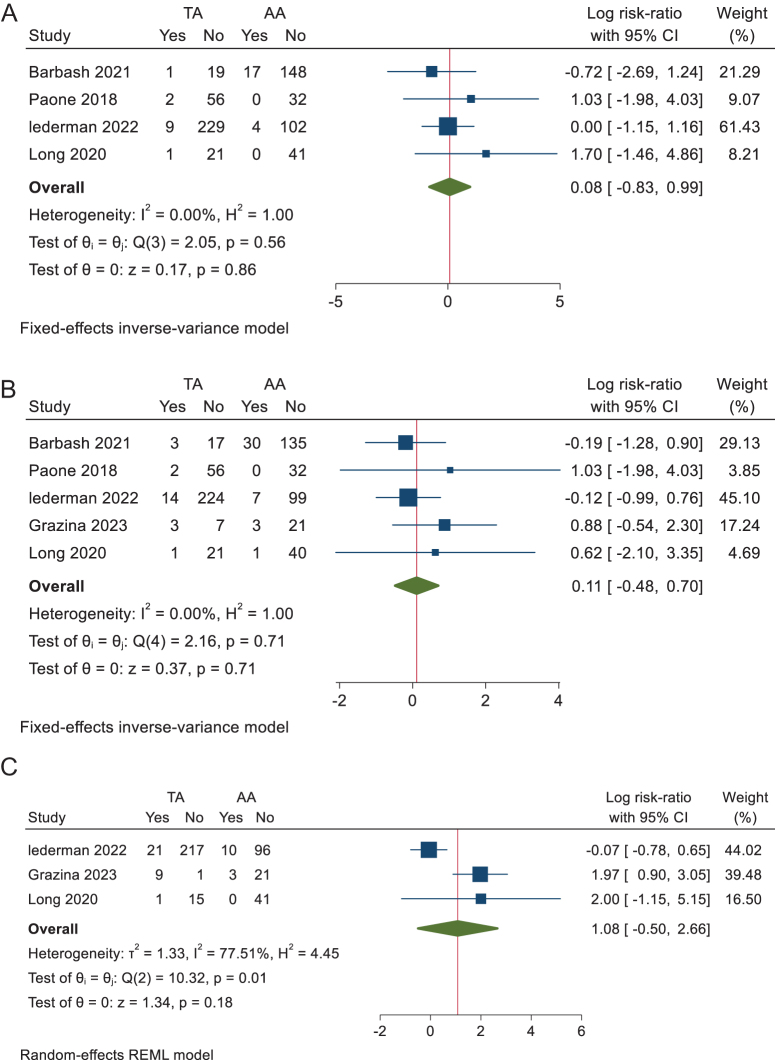
(A) Forest plot of intra-hospital death. (B) Forest plot of 30 days all-cause mortality. (C) Forest plot of 1 year all-cause mortality.

Our analysis of intra-hospital all-cause mortality included four studies comparing transcaval to alternative accesses, shows no statistically significant difference in the rates of mortality compared to other alternative accesses with an RR of 0.08 (95% CI −0.83 to 0.99, *P* = 0.86) (Fig. 1A). No heterogeneity was observed (I^2^ = 0%, *P* = 0.56).

ii. 30 days all-cause mortality

Our analysis of 30-day all-cause mortality included five studies comparing transcaval to alternative accesses shows no statistically significant difference in the rates of mortality compared to other alternative accesses with a RR of 0.11 (95% CI −0.48 to 0.7, *P* = 0.71) (Fig. 1B). No heterogeneity was observed (I^2^ = 0%, *P* = 0.71).

iii. 1-year all-cause mortality

Three studies were included in the analysis of 1-year All-cause mortality, showing no statistically significant difference in the mortality rates between transcaval and other alternative accesses with an RR of 1.08 (95% CI −0.50 to 2.66, *P* = 0.18) (Fig. 1C). Heterogeneity was high (I^2^ = 77.51%, *P* = 0.01), which was solved by omitting Lederman *et al* 2022 (Supplementary Figure 6, available at: http://links.lww.com/MS9/A775).

Morbidity
  Length of Intra hospital stay

**Figure 2.**
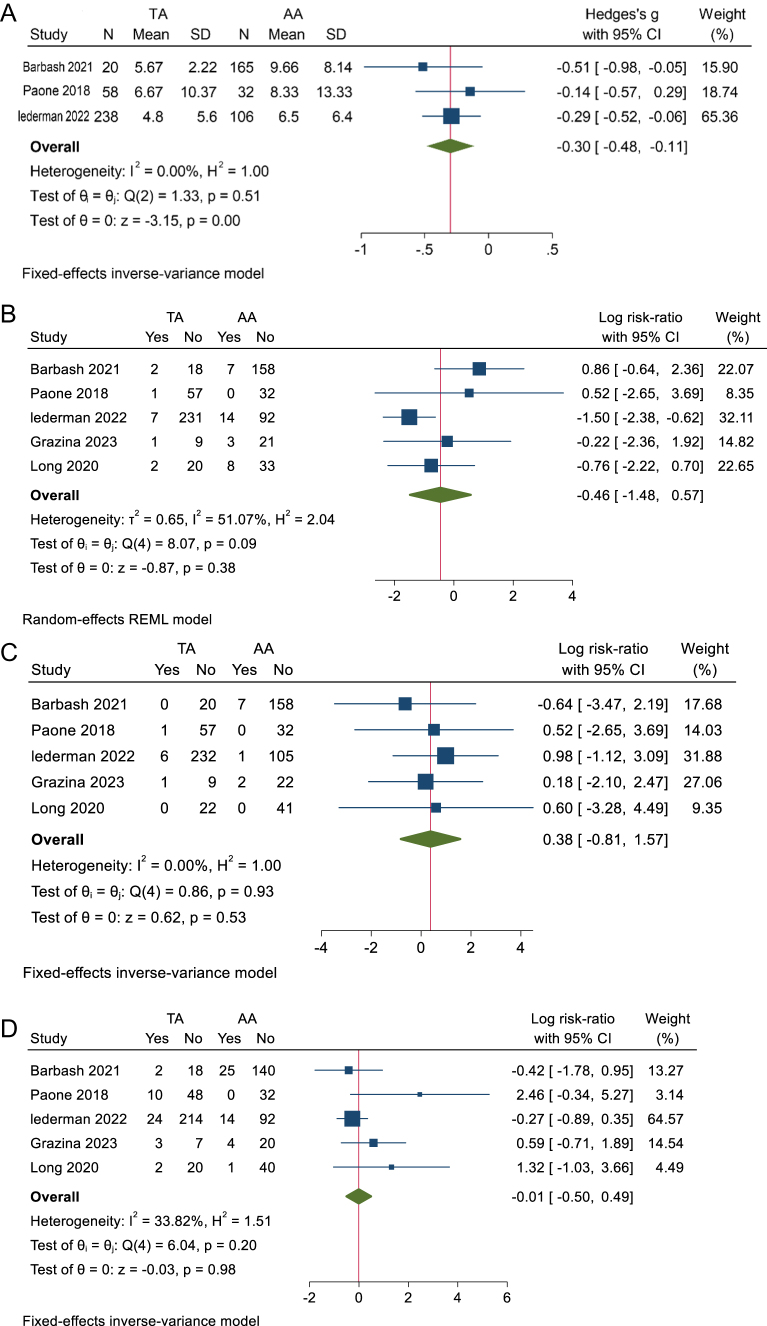
(A) Forest plot of length of intra hospital stay. (B) Forest plot of stroke or TIA. (C) Forest plot of vascular access complications. (D) Forest plot of major bleeding.

Three studies showed no statistically significant difference in the rates of intra-hospital stay between transcaval and other alternative accesses with an MD of −0.3 (95% CI −0.48 to −0.11, *P* = 0.0) (Fig. 2A). No heterogeneity was observed (I^2^ = 0%, *P* = 0.51).

ii. Stroke or TIA

The analysis of postoperative stroke included five studies comparing transcaval against other alternative accesses, showing no statistically significant difference with an RR of −0.46 (95% CI −1.48 to 0.57, *P* = 0.38) (Fig. 2B). The heterogeneity was high (I^2^ = 51.07%, *P* = 0.09), which was solved by removing Barbash *et al* 2021 (Supplementary Figure 7, available at: http://links.lww.com/MS9/A775).

iii. Vascular access complications

The analysis of vascular access complications included five studies comparing transcaval against other alternative accesses. Transcaval had no statistically significant difference in the rates of vascular complications compared to other alternative accesses with an RR of 0.38 (95% CI −0.81 to 1.57, *P* = 0.53) (Fig. 2C). No heterogeneity was observed in (I^2^ = 0%, *P* = 0.93).

iv. Major bleeding

The analysis of major bleeding included five studies comparing transcaval against other alternative accesses. Transcaval had no statistically significant difference in the rates of major bleeding compared to other alternative accesses with a RR of −0.01 (95% CI −0.50 to 0.49, *P* = 0.98). There was no heterogeneity among the included studies (I^2^ = 33.82%, *P* = 0.2) (Fig. 2D).

v. Acute kidney injury

**Figure 3.**
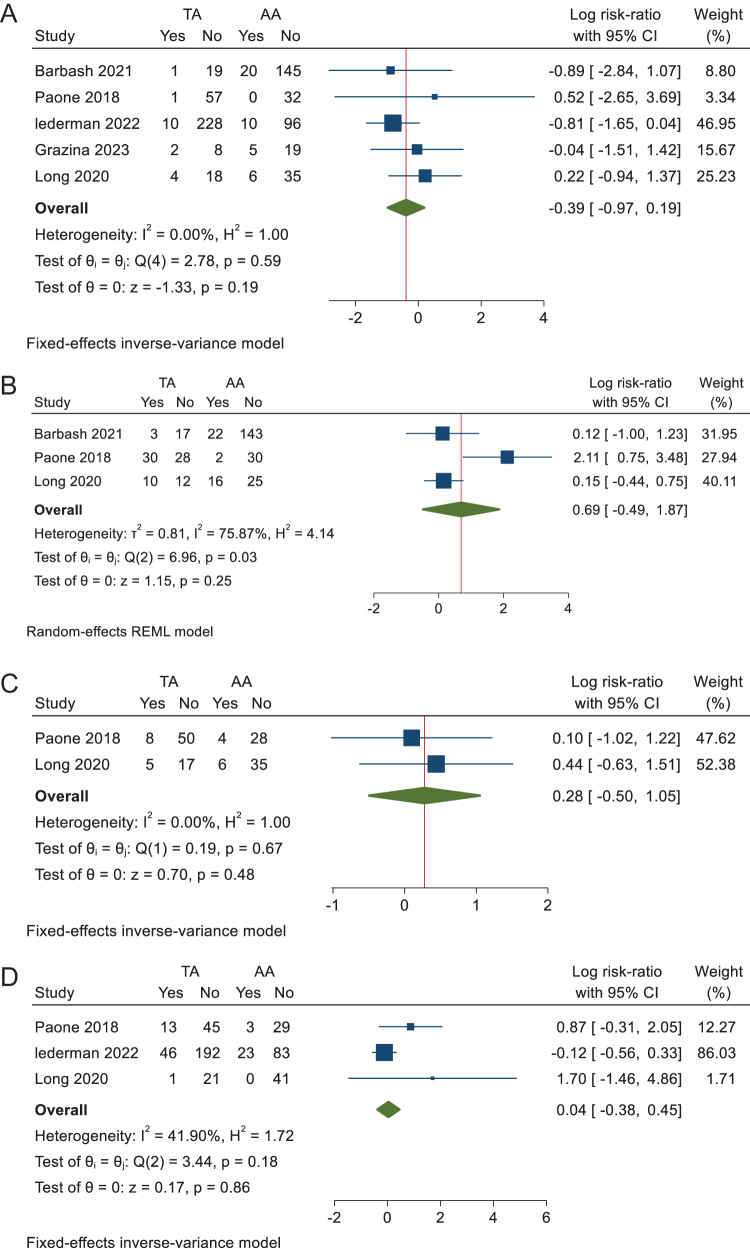
(A) Forest plot of acute kidney injury. (B) Forest plot of new pacemaker implantation. (C) Forest plot of readmission within 30 days. (D) Forest plot of transfusion need.

The analysis of acute kidney injury included five studies with no statistically significant difference between transcaval and other alternative accesses, with a RR of −0.39 (95% CI −0.97 to 0.19, *P* = 0.19) (Fig. 3A). No heterogeneity was observed (I^2^ = 0%, *P* = 0.59).

vi. New pacemaker implantation

The analysis of new pacemaker implantation included three studies with no statistically significant difference between the two groups with a RR of 0.69 (95% CI −0.49 to 1.87, *P* = 0.25) (Fig. 3B). Heterogeneity was high (I^2^ = 71%, *P* = 0.03), which was solved by removing Paone *et al* 2018 (Supplementary Figure 8, available at: http://links.lww.com/MS9/A775).

vii. Readmission within 30 days

Only two studies reported readmission within 30 days, showing no statistically significant difference between transcaval and other alternative accesses with an RR of 0.28 (95% CI −0.50 to 1.05, *P* = 0.48) (Fig. 3C). No heterogeneity was observed (I^2^ = 0%, *P* = 0.67).

viii. Transfusion needs

Three studies reported blood transfusion rates. Our analysis showed no statistically significant difference between transcaval and other alternative access groups, with an RR of 0.04 (95% CI −0.38 to 0.45, *P* = 0.86) (Fig. 3D). No significant heterogeneity was observed (I^2^ = 41.9%, *P* = 0.18).

#### Single-group analysis

- Mortality
  - 30 days all-cause mortality

**Figure 4.**
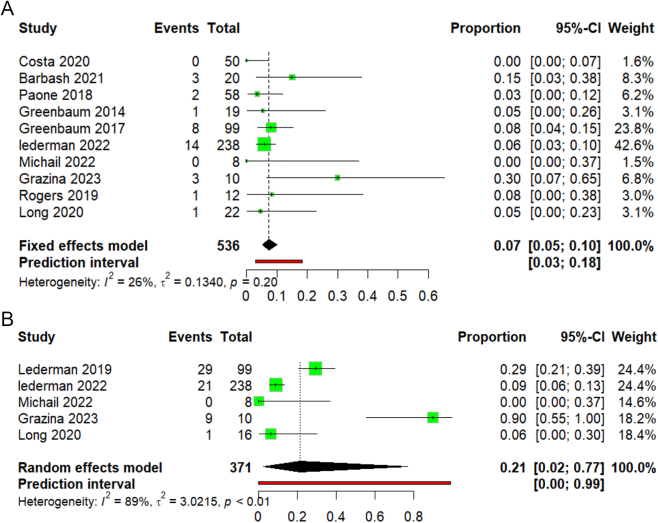
(A) Forest plot of 30 days all-cause mortality. (B) Forest plot of 1-year all-cause mortality.

Ten studies reported the 30-day all-cause mortality rate. The pooled mortality rate across all studies was 7% (95% CI 5–10), as shown in (Fig. 4A). Heterogeneity was observed (I^2^ = 26%, *P* = 0.2).

ii. 1-year all-cause mortality

Five studies reported 1-year all-cause mortality rate. The pooled mortality rate across studies was 21% (95% CI 2–77), as shown in (Fig. 4B). Studies were highly heterogeneous (I^2^ = 89%, *P* < 0.01). By leaving out analysis, we found that removing the study Lederman 2022 has the lowest heterogeneity by I^2^ = 78% (Supplementary Figure 9, available at: http://links.lww.com/MS9/A775).

- Morbidity
  - Length of hospital stay

**Figure 5.**
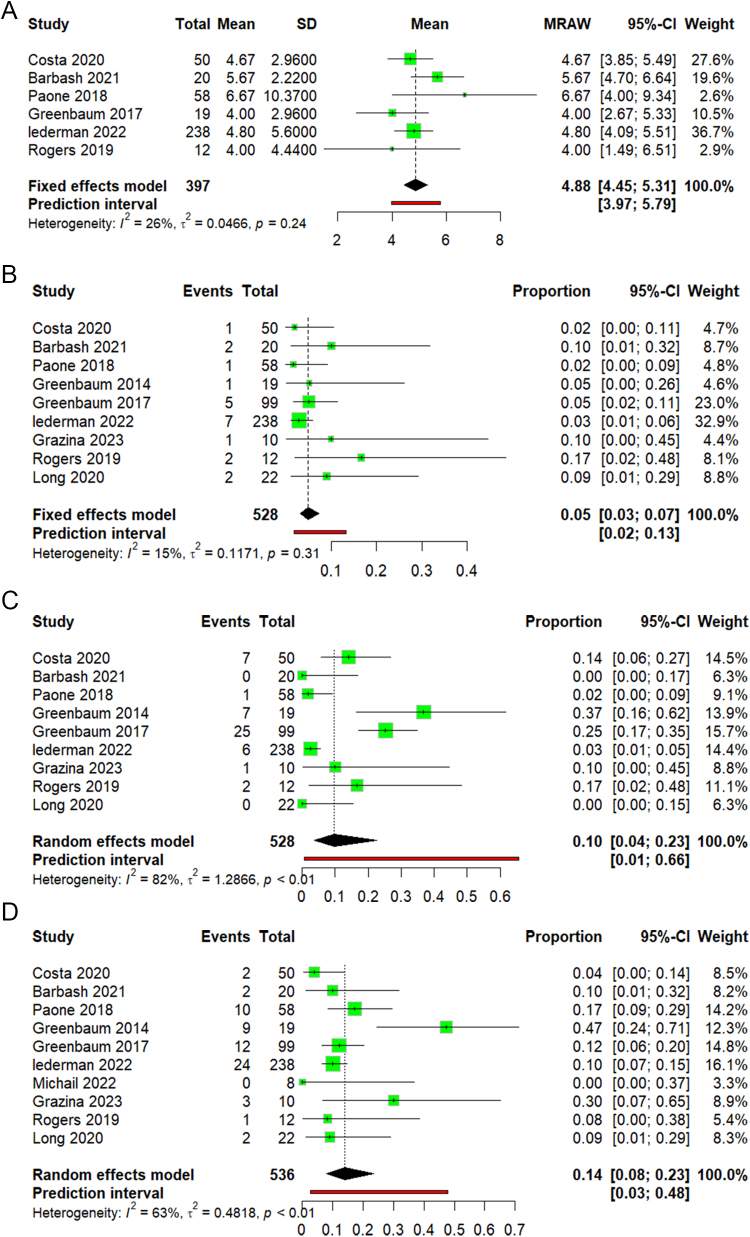
(A) Forest plot of length of hospital stay. (B) Forest plot of stroke. (C) Forest plot of vascular complications. (D) Forest plot of major bleeding.

Six studies reported the mean length of stay in the hospital after the operation. The pooled hospital stay across all studies was 4.88 (95% CI 4.45–5.31), as shown in (Fig. 5A). No heterogeneity was observed (I^2^ = 26%, *P* = 0.24).

ii. Stroke

Regarding postoperative stroke, an analysis of nine studies showed a pooled stroke rate across all studies equal to 5% (95% CI 3–7), as shown in (Fig. 5B). No heterogeneity was observed (I^2^ = 15%, *P* = 0.31).

iii. Vascular complications

In our analysis, nine studies reporting vascular complication rates were included. The pooled vascular complication rate across all studies was 10% (95% CI 4–23), as shown in (Fig. 5C). Studies were highly heterogeneous (I^2^ = 82%, *P* < 0.01). By leaving out analysis, we found that removing the study Lederman 2022 has the lowest heterogeneity by I^2^ = 63% (Supplementary Figure 10, available at: http://links.lww.com/MS9/A775).

iv. Major bleeding

Ten studies reported the rate of major bleeding. The pooled major bleeding rate across all studies was 14% (95% CI 8–23), as shown in (Fig. 5D). Heterogeneity was observed (I^2^ = 63%, *P* < 0.01).

By leaving out analysis, we found that removing the study Greenbaum 2014 has the lowest heterogeneity by I^2^ = 5% (Supplementary Figure 11, available at: http://links.lww.com/MS9/A775).

v. Acute kidney injury

**Figure 6.**
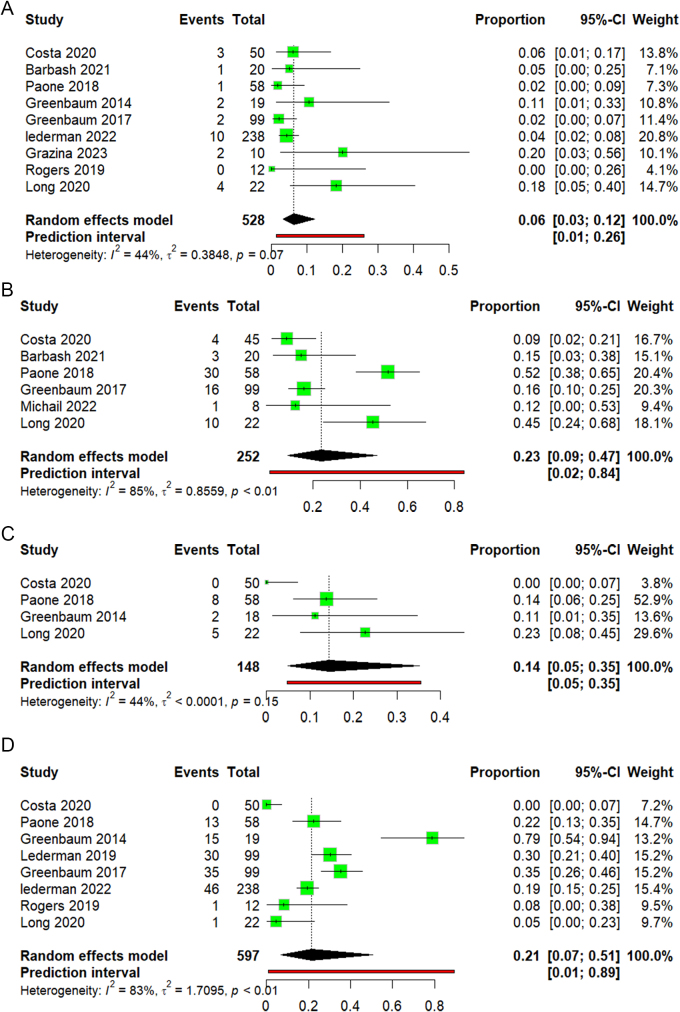
(A) Forest plot of acute kidney injury. (B) Forest plot of new pacemaker implantation. (C) Forest plot of readmission within 30 days. (D) Forest plot of blood transfusion.

In our analysis, nine studies reporting acute kidney injury rates were included. The pooled acute kidney injury rate across all studies was 6% (95% CI 3–12), as shown in (Fig. 6A). No heterogeneity was observed (I^2^ = 44%, *P* = 0.07). By leaving one out, we found that removing the study Long 2020 has the lowest heterogeneity by I^2^ = 17% (Supplementary Figure 12, available at: http://links.lww.com/MS9/A775).

vi. New pacemaker implantation

Regarding new pacemaker implantation, an analysis of six studies showed a pooled implantation rate across all studies equal to 23% (95% CI 9–47), as shown in (Fig. 6B). Significant heterogeneity was observed (I^2^ = 85%, *P* < 0.01). By leaving out analysis, we found that removing study Paone 2018 has the lowest heterogeneity by I^2^ = 68% (Supplementary Figure 13, available at: http://links.lww.com/MS9/A775).

vii. Readmission within 30 days

Four studies reported a readmission rate. The pooled readmission rate across both studies was 14% (95% CI 5–35), as shown in (Fig. 6C). Non-significant heterogeneity was observed (I^2^ = 44%, *P* = 0.15). By leaving out an analysis, we found that removing the study Costa 2020 has the lowest heterogeneity by I^2^ = 0% (Supplementary Figure 14, available at: http://links.lww.com/MS9/A775).

viii. Blood transfusion

Eight studies reported blood transfusion rates. The pooled blood transfusion rate across all studies was 21% (95% CI 7–51), as shown in (Fig. 6D). Studies were highly heterogeneous (I^2^ = 83%, *P* < 0.01). By leaving out an analysis, we found that removing the study Greenbaum 2014 has the lowest heterogeneity by I^2^ = 73% (Supplementary Figure 15, available at: http://links.lww.com/MS9/A775).

## Discussion

The present systemic review and meta-analysis address the importance of transcaval access for TAVR in patients unsuitable for traditional transfemoral access. Our analysis showed that among patients with contraindications to the trans-femoral access, the transcaval approach is as safe and effective as other approaches (trans-axillary and trans-apical) regarding mortality, vascular complications, major bleeding, permanent pacemaker implantation, transfusion need, acute kidney injury and length of hospital stay. The need for non-TF TAVI procedures has decreased due to improved introducer sheaths and smaller transcatheter heart valve delivery systems. However, a particular group of patients with specific anatomical considerations will always remain, and they can only be treated by alternative access approaches.

In the study conducted by Costa *et al*, the incidence of life-threatening bleeding and major vascular complications associated with the transcaval access route for TAVR were evaluated using the Valve Academic Research Consortium-2 (VARC-2) criteria. The findings revealed that life-threatening bleeding occurred in 4% of patients, while major vascular complications were observed in 10% of cases^[23]^. These rates are comparable to those reported in the largest cohort of transcaval TAVI procedures in the United States, which included 100 patients and documented life-threatening bleeding and major vascular complication rates of 7% and 13%, respectively^[19]^.

Furthermore, Costa *et al* reported that no major vascular complications or bleeding events were observed after patient discharge, suggesting a favorable short-term safety profile post-procedure. However, within their cohort, two significant vascular complications were directly attributed to the embolization of an Angio-Seal™ collagen plug, a device used to close the arterial access site. These complications necessitated surgical intervention, and tragically, one patient succumbed to acute kidney injury and sepsis as a result.

Based on these observations, Costa and colleagues concluded that suture-based closure devices may offer a safer alternative to collagen-based devices, particularly in patients with severe peripheral vascular disease. The rationale behind this recommendation is that suture-based devices might reduce the risk of device-related complications, such as embolization, thereby enhancing the overall safety and efficacy of the transcaval access approach^[23]^. This conclusion underscores the critical importance of selecting appropriate closure mechanisms to minimize adverse events and improve patient outcomes in transcaval TAVI procedures.

Greenbaum *et al*^[19]^ documented that among 100 consecutive patients who underwent TAVI via the transcaval access route, procedural success in both access and closure was achieved in 99% of cases, with no reported procedural mortality or need for surgical intervention. The transcaval approach, originally developed at the National Institutes of Health^[24]^, was designed to address challenges posed by severely diseased or hostile iliofemoral arteries that cannot accommodate the delivery of large-bore devices and sheaths. This technique leverages the principles of electrosurgery and relies on the physiological differences in pressure gradients between three distinct compartments: the inferior vena cava, which has the lowest pressure; the abdominal aorta, which has the highest pressure; and the retroperitoneal space, where both vessels are located. The retroperitoneal pressure is intermediate, higher than the vena cava but lower than the aorta. Based on these hemodynamic principles, any bleeding from the aorta during the procedure tends to flow into the vena cava, the compartment with the lowest pressure, rather than into the retroperitoneal space. This creates a temporary aorto-venous fistula rather than massive retroperitoneal hemorrhage, providing the operator with the opportunity to achieve hemostasis while maintaining the patient’s hemodynamic stability^[25]^.

Costa *et al* found that about 50% of patients had a residual aortocaval shunt immediately after completion of the TAVI procedure. However, these shunts had no hemodynamic significance, and none had any re-hospitalization due to bleeding or dyspnoea within 30 days of discharge^[23]^. Furthermore, in another study by Greenbaum and colleagues, more than 80% of the remaining aortocaval shunts close spontaneously by 42 days (range 7-189 days)^[20]^.

Lederman and colleagues found that 77 (93%) of fistulas were proven occluded by 12 months with fistula patency had no association with overall survival (*P* = 0.37) nor with heart failure admissions (*P* = 0.30)^[17]^. Another study showed that the aorto-caval fistula occluded in most patients (72%) by 30 days, as proven by computed tomography^[21]^.

In a study conducted by Greenbaum *et al*, one patient developed severe but asymptomatic thrombocytopenia, with a platelet count of 24 000 cells/µL, which resolved spontaneously following the closure of the caval-aortic tract. Additionally, eight other patients experienced a greater than 50% reduction in platelet counts^[20]^. This phenomenon may be considered a physiological response associated with the presence of an aorto-caval shunt. Similar thrombocytopenia has been reported following device closure of a patent ductus arteriosus^[26]^, which was attributed to mechanical platelet injury.

In the context of transcaval TAVI, the observed thrombocytopenia may result from platelet consumption due to microvascular bleeding or persistent residual aorto-caval shunting^[27]^. Further investigation is warranted to better understand the mechanisms underlying this hematological response and to assess its clinical significance.

Evidence regarding the safety and efficacy of alternative access approaches for TAVI remains inconsistent across studies. A large-scale analysis derived from the Society of Thoracic Surgeons/American College of Cardiology (STS/ACC) Transcatheter Valve Therapies Registry^[28]^ indicated that non-transfemoral TAVI access is associated with a higher risk of 1-year mortality. Specifically, several studies have demonstrated that the transapical approach is linked to significantly increased mortality rates and a higher incidence of complications, including stroke, cardiac tamponade, and the need for permanent pacemaker implantation^[29,30]^.

Conversely, other studies have reported comparable outcomes between transfemoral and transapical approaches, suggesting that the observed differences in outcomes may be partially attributed to poorer baseline characteristics of patients undergoing the transapical approach, as well as variations in operator experience with the technique^[31-33]^. These findings highlight the need for further investigation to clarify the role of alternative access routes and to optimize patient selection and procedural strategies.

In their study, Taramasso and colleagues^[34]^ determined that the trans-axillary approach carries long-term risks comparable to those associated with the transapical method. These findings highlight the urgent need for the development of innovative access strategies that do not elevate mortality rates or increase vascular complications, especially for patients with compromised iliofemoral anatomy or disease. The safety and efficacy of transcaval access routes continue to be a topic of debate within the medical community. For example, Grazina *et al*^[20]^ demonstrated that the transfemoral access route yielded superior outcomes compared to alternative access methods concerning mortality rates, postoperative acute kidney injury, 30-day stroke incidence, and major bleeding events. However, these outcome disparities were partly attributed to differences in baseline characteristics among the patient populations studied. Furthermore, Grazina and colleagues found that the 1-year mortality rate was similar between patients undergoing TAVI via alternative routes and those receiving medical management alone, raising concerns about the overall benefit and necessity of these alternative procedural approaches^[20]^.

In a study conducted by Paone *et al*, the transcaval approach was evaluated in 58 patients and compared to both the standard transfemoral and transcarotid access methods. The results demonstrated that short-term and long-term outcomes were similar across all three groups, despite the transcaval cohort having a higher prevalence of baseline comorbidities^[15]^. This indicates that the transcaval approach may be as effective as traditional and alternative access routes, even in patients with more complex health profiles. Additionally, Long *et al* conducted a more recent investigation involving 22 patients, comparing the transcaval approach to the subclavian access route. Their findings revealed that the subclavian group experienced higher rates of early stroke and an increased need for pacemaker implantation, while there were no significant differences in mortality between the two approaches^[7]^. These studies collectively suggest that the transcaval approach not only offers comparable efficacy to other access methods but may also reduce certain procedural complications. The ability to achieve similar outcomes in populations with greater comorbidities further underscores the potential of the transcaval route as a viable alternative in TAVI, particularly for patients with challenging vascular anatomies.

In a retrospective analysis utilizing a large registry, Lederman *et al*^[17]^ found that the transcaval access route for TAVI was associated with significantly lower rates of stroke (OR 0.20; *P* = .014) and comparable rates of bleeding (OR 0.66; *P* = .38) when compared to the transaxillary approach. These findings suggest that transcaval access may offer a safer alternative with respect to neurological complications without increasing hemorrhagic risks. Furthermore, when compared to other alternative access routes such as carotid, subclavian, and axillary artery approaches, the transcaval method presents several procedural advantages. These advantages include enhanced operator familiarity due to the utilization of the standard right groin puncture site, which is a common entry point in many vascular procedures. Additionally, the transcaval approach involves a less tortuous sheath trajectory, thereby simplifying the procedural mechanics and potentially reducing procedure time. The method also minimizes the risk of brachial plexus injury, a notable complication associated with upper extremity access routes, and eliminates the need for surgical dissection, thereby reducing operative trauma and recovery time^[19]^.

Severe aortic stenosis, if left untreated, carries a grave prognosis, with mortality rates reaching approximately 50% within two years. This underscores the critical importance of selecting the most appropriate access route for TAVI, especially in high-risk patient populations. Optimal access strategies are essential not only for improving clinical outcomes but also for enhancing procedural safety and reducing complication rates. As the population of patients requiring TAVI continues to grow, particularly among those with complex vascular anatomies or comorbid conditions, the transcaval approach emerges as a promising alternative. Future research should focus on larger, randomized controlled trials to further validate these findings and to establish standardized protocols that maximize the benefits of transcaval access while mitigating potential risks. By refining access techniques and expanding the evidence base, clinicians can better tailor TAVI procedures to individual patient needs, ultimately improving survival rates and quality of life for those afflicted by severe aortic stenosis.

### Strengths and limitations

Although this study represents the first comprehensive review and meta-analysis evaluating transcaval TAVI access as an alternative route, it has several limitations. First, all included studies were observational with a limited number of patients, and the transcaval group often had worse baseline characteristics, potentially underestimating the efficacy of this approach. Additionally, most studies reported outcomes over short follow-up periods, limiting the assessment of long-term performance. The analysis also included only a small number of double-arm studies, and despite an extensive literature search, no additional double-arm studies were identified, restricting our ability to perform subgroup analyses based on specific comparators. However, we have analyzed double-arm studies separately, then pooled then in another analysis with the single-arm studies. Furthermore, the limited number of studies for each outcome made it infeasible to conduct a meta-regression analysis to adjust for potential confounding factors. Pooling different approaches into a single comparator category may have introduced heterogeneity, which could impact the interpretation of the results. While efforts were made to address these limitations, future studies with larger sample sizes and direct comparisons are essential to validate our findings and provide more robust conclusion. Furthermore, we recommend and call for conducting clinical trials that compare the standard transfemoral approach to alternative routes such as transcaval in patients with hostile iliofemoral anatomy and severe vascular disease. These trials should recruit sufficient participants to gain the best clinical evidence regarding the appropriate approach in those cohorts of patients.

## Conclusion

Transcaval access proved to be a safe and effective alternative TAVI access route for high-risk patients with severe aortic stenosis with prohibitive risk for transfemoral and transaxillary/subclavian access. It is expected that the outcomes of this TAVI approach will have a positive learning curve over time as centers gain more experience with the technique and a dedicated caval-aortic closure device becomes available.

## Data Availability

None.

## Research registration unique identifying number (UIN)

Our study was registered on the Open Science Framework with DOI: 10.17605/OSF.IO/WUASM.
